# Ablation of *Tmcc2* Gene Impairs Erythropoiesis in Mice

**DOI:** 10.3390/ijms23095263

**Published:** 2022-05-09

**Authors:** Ranju Kumari, Tomasz M. Grzywa, Milena Małecka-Giełdowska, Karolina Tyszkowska, Robert Wrzesień, Olga Ciepiela, Dominika Nowis, Piotr Kaźmierczak

**Affiliations:** 1 Centre of New Technologies, University of Warsaw, Banacha 2C, 02-097 Warsaw, Poland; r.kumari@cent.uw.edu.pl; 2 School of Molecular Medicine, Medical University of Warsaw, Żwirki i Wigury 61, 02-091 Warsaw, Poland; 3 Department of Immunology, Medical University of Warsaw, Nielubowicza 5, 02-097 Warsaw, Poland; grzywa.t@gmail.com (T.M.G.); dominika.nowis@wum.edu.pl (D.N.); 4 Doctoral School, Medical University of Warsaw, Żwirki i Wigury 61, 02-091 Warsaw, Poland; 5 Laboratory of Experimental Medicine, Medical University of Warsaw, Nielubowicza 5, 02-097 Warsaw, Poland; 6 Department of Laboratory Medicine, Medical University of Warsaw, Banacha 1A, 02-097 Warsaw, Poland; milena.malecka@wum.edu.pl (M.M.-G.); olga.ciepiela@wum.edu.pl (O.C.); 7 Central Laboratory of Experimental Animal, Centre for Preclinical Research, Medical University of Warsaw, Banacha 1B, 02-097 Warsaw, Poland; karolina.tyszkowska@wum.edu.pl (K.T.); robert.wrzesien@wum.edu.pl (R.W.)

**Keywords:** erythropoiesis, anemia, polycythemia, congenital dyserythropoietic anemia, *TMCC2*, knockout mice, erythrocyte, erythroblasts, enucleation

## Abstract

(1) Background: Transcriptomic and proteomic studies provide a wealth of new genes potentially involved in red blood cell (RBC) maturation or implicated in the pathogenesis of anemias, necessitating validation of candidate genes in vivo; (2) Methods: We inactivated one such candidate, transmembrane and coiled-coil domain 2 (*Tmcc2*) in mice, and analyzed the erythropoietic phenotype by light microscopy, transmission electron microscopy (TEM), and flow cytometry of erythrocytes and erythroid precursors; (3) Results: *Tmcc2^−/−^* pups presented pallor and reduced body weight due to the profound neonatal macrocytic anemia with numerous nucleated RBCs (nRBCs) and occasional multinucleated RBCs. *Tmcc2^−/−^* nRBCs had cytoplasmic intrusions into the nucleus and double membranes. Significantly fewer erythroid cells were enucleated. Adult knockouts were normocytic, mildly polycythemic, with active extramedullary erythropoiesis in the spleen. Altered relative content of different stage CD71^+^TER119^+^ erythroid precursors in the bone marrow indicated a severe defect of erythroid maturation at the polychromatic to orthochromatic transition stage; (4) Conclusions: *Tmcc2* is required for normal erythropoiesis in mice. While several phenotypic features resemble congenital dyserythropoietic anemias (CDA) types II, III, and IV, the involvement of *TMCC2* in the pathogenesis of CDA in humans remains to be determined.

## 1. Introduction

The molecular determinants of erythropoiesis, a process of cellular proliferation and differentiation that leads to the production of red blood cells (RBCs), have been the subject of much research [1]. Transcriptomic, proteomic, and chromatin state analyses have revealed thousands of genes or proteins that are regulated during this process, including genes already known to be critically important in the experimental setting and in hematological practice. The role of the remaining majority remains to be validated and explored, as they are candidates for novel causative genes in hereditary disturbances of erythropoiesis and potential targets for therapies. Among the most interesting leads is the highly enhanced expression of human *TMCC2* (transmembrane and coiled-coil domains 2), mouse *Tmcc2* genes, and *TMCC2* proteins during terminal differentiation of erythroblasts [2,3,4]. *TMCC2* exists in two major isoforms that originate from alternative promoters. Interference with the erythroid specific isoform of *TMCC2* affects cellular morphology and reduces efficiency of erythroblast maturation in vitro [4]. Infection of human erythroid cells with lentiviral vectors carrying *TMCC2* specific shRNA constructs leads to three clear effects: (i) reduced proliferation seen at 3, 5, and 8 days after infection; (ii) increased frequency of apoptotic cells that are positive for annexin V, from nearly 5% in controls to between 15% and 20% in *TMCC2* knockdown cultures; and (iii) disrupted morphology of *TMCC2* knockdown cells, pointing to aberrant maturation, with remnants of dead cells visible in the microscopic images. However, the role of TMMC2 in erythropoiesis in vivo remains unknown. A spliced early postnatal spleen library expressed sequence tag (AK165309) indicates that this isoform is expressed in an erythropoietic tissue in mice. 

Our interest in *TMCC2* was further driven by the fact that the cellular and physiological function of this protein in vertebrates remained unknown. The homolog of *TMCC2* was first characterized in Drosophila melanogaster as a neuronal protein Dementin (Dmtn) involved in the development of the brain, APP processing, and the formation of amyloid deposits [5,6]. However, flies only have one gene of the Tmcc family whilst mammals have three such genes, making any parallels uncertain. Moreover, flies do not have erythrocytes. Thus, a vertebrate model was necessary to test whether *TMCC2* was indeed required for erythropoiesis.

## 2. Results

To investigate the function of *TMCC2* in vivo, we generated a *Tmcc2* knockout mouse line by Clustered Regularly Interspaced Short Palindromic Repeats/CRISPR associated protein 9 (CRISPR/Cas9)-mediated deletion of 11 nucleotides (c.835_845del11b) that introduces a stop codon (ENSMUST00000045473.15 p.279X) in all *Tmcc2* isoforms. We opted for complete gene inactivation as opposed to a conditional myeloid specific promoter driven knockout in order to study the full extent of the phenotype and to avoid the risk of missing some aspects of *TMCC2* function due to potential incomplete recombination of a conditional allele. Simplicity and better cost effectiveness of complete knockouts were additional factors that we took into account.

Full length *TMCC2* was detected in brain extracts from heterozygous P5 (5 days after birth) pups but not homozygous mutants (Figure S1A–D, Materials and Methods).

**Figure 1:**
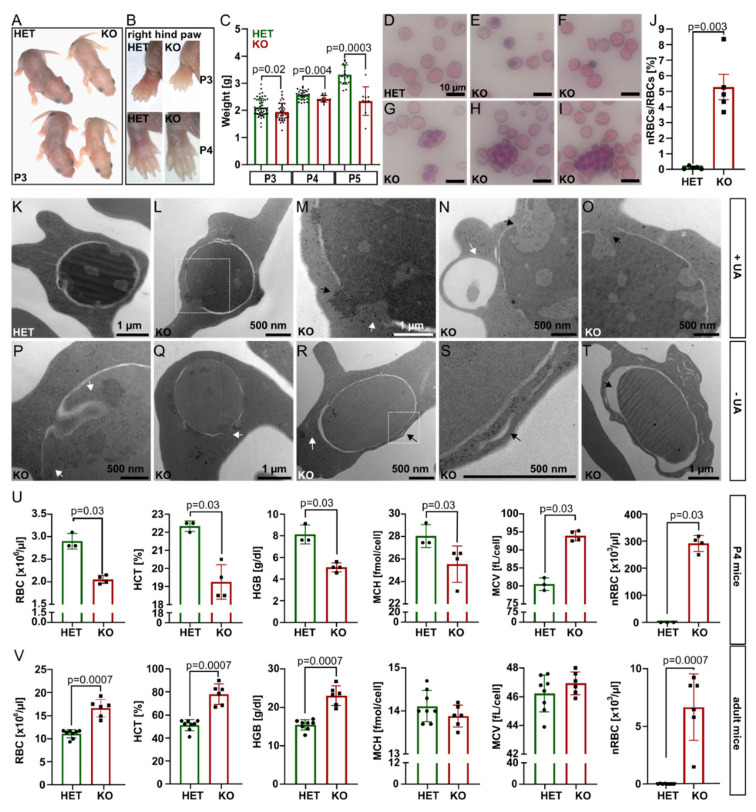
*Tmcc2* knockout mouse apparent phenotype. (**A**) *Tmcc2^−/−^* pup appearance, (**B**) pallor of the limbs. (**C**) Reduced weight of *Tmcc2^−/−^* pups (P3: N_HET_ = 52, N_KO_ = 36; P4: N_HET_ = 30, N_KO_ = 18; P5: N_HET_ = 18, N_KO_ = 9; *p*-values by *t*-test). Blood smear (P3): (**D**) normal RBCs in heterozygotes, (**E**–**I**) nRBCs in knockouts, (**G**–**I**) multinucleated erythroblasts, (**J**) nRBC fraction (N_HET_ = 5, N_KO_ = 5) (see Figure S2E–G). TEM of (**K**) control and (**L**–**T**) *Tmcc2^−/−^* nRBCs (P2), (L–P) nuclear membrane invagination, rupture, cytoplasmic and ribosomal intrusions; (R, S) double membranes; (T) dilated perinuclear cisterns; (**N**,**R**) autophagosomes; (**T**) numerous membranous cisterns. (**U**–**V**) CBC in (**U**) P4 (N_HET_ = 3, N_KO_ = 4, either sex) and (**V**) adult mice (N_HET_ = 8, N_KO_ = 6, female). *p*-values by Mann–Whitney test.

At P1–P3, *Tmcc2^−/−^* pups were pale, smaller, and lighter by 7.9% (P3) to 29.7% (P5) compared to the heterozygotes. By P4, the discoloration was restricted to the ear lobes and paws (Figure 1A–C). Adult littermates were of comparable weight (Figure S1E). We suspected neonatal anemia.

Interestingly, the knockout blood smears showed a significant proportion of nucleated RBCs (nRBCs), occasional pyrenocytes, and giant multinucleated cells with up to 12 nuclei (Figure 1D–J and Figure S2A–G), akin to a group of congenital dyserythropoietic anemias (CDAs). Four CDA types are recognized based on clinical findings and genetics [7]. Mutations in codanin (CDAN1) [8] and CDAN1-interacting-nuclease (CDIN1) [9] cause CDA I; *SEC23B* [10], *KIF23* [11], and *KLF1* [12] are mutated in CDA II-IV, and *GATA1* [13] in a variant of CDA. Transmission electron microscopy (TEM) of *Tmcc2^−/−^* P2 blood samples (Figure 1K–T and Figure S3) revealed nRBCs at various maturation stages with such defects as dilated perinuclear space, the double membranes typical for CDA II, and nuclear cytoplasmic intrusions reminiscent of CDA IV.

Complete blood count (CBC) at P4 confirmed severe macrocytic anemia in the knockout pups (Figure 1U and Figure S2H–J). The RBC count, hematocrit (HCT), hemoglobin concentration (HGB), and mean corpuscular hemoglobin concentration (MCHC) were all drastically reduced, while the mean corpuscular volume (MCV) and the fraction of macrocytes were increased. Most strikingly, the mean nRBC count (>291 × 10^3^/µL) was elevated by 1742 times. Intriguingly, adult *Tmcc2^−/−^* mice had normal-sized erythrocytes and increased RBC count, hemoglobin content, and hematocrit. Scarce nRBCs were still present (Figure 1V). The bilirubin level was low at both ages, which indicates that hemolysis is unlikely to contribute to the phenotype. Iron, ferritin, and serum biochemistry in adults were normal (Figure S2L–S).

**Figure 2:**
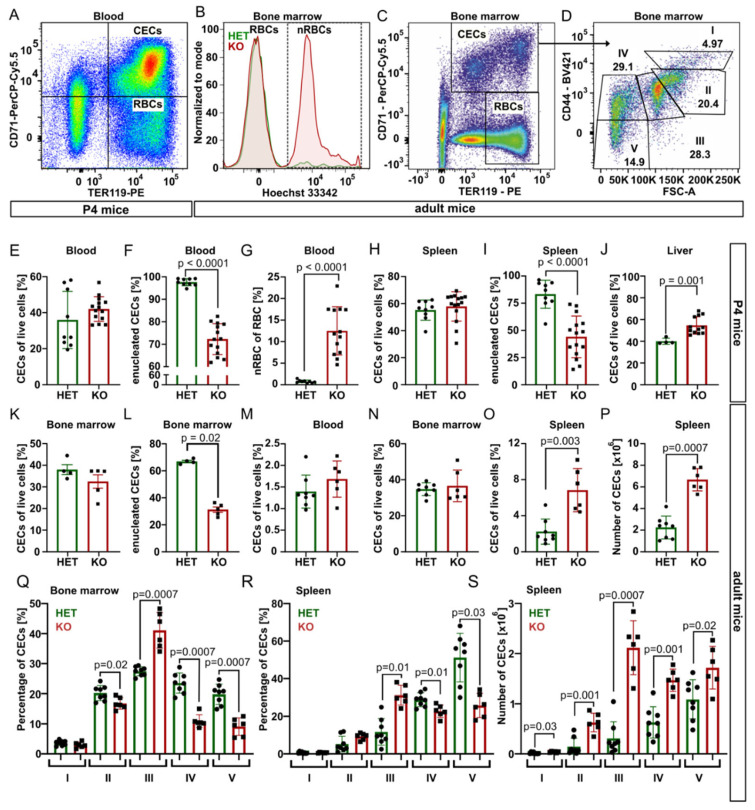
Flow cytometry analysis of CECs and RBCs. Experimental set-up: (**A**) Gating of CECs and erythrocytes (RBCs). (**B**) Hoechst 33342 signal defines nRBCs. (**C**) Gating strategy, (**D**) maturation stages of CECs [14]. Results, pups (either sex): (**E**–**G**) abundance and enucleation status of CECs and nRBCs in blood (N_HET_ = 9, N_KO_ = 14); (**H**,**I**) spleen (N_HET_ = 9, N_KO_ = 15); (**J**) liver, non-parenchymal (N_HET_ = 4, N_KO_ = 12). Adults (female): (**K**,**L**) bone marrow, young adult (N_HET_ = 4, N_KO_ = 5); (M–S) 7–8 months old (N_HET_ = 8, N_KO_ = 6); (**M**) blood; (**N**) bone marrow; (**O**) spleen; and (**P**) absolute number of CECs in spleen. Maturation of CECs: percentage of different immature stages in the (**Q**) bone marrow and (**R**) the spleen and (**S**) absolute numbers in the spleen: proerythroblasts (I), basophilic (II), polychromatic (III), orthochromatic (IV), and reticulocytes (V). *p*-values by Mann–Whitney test.

Next, we took advantage of specific surface markers CD71 (Transferrin receptor 1) and TER119 (murine erythroid cell surface marker coded by the *Ly76* gene) and analyzed using flow cytometry CD71^+^TER119^+^ erythroid progenitor and precursor cells (CECs) and mature erythrocytes (CD71^−^TER119^+^) from blood, bone marrow, spleen, and liver samples of P4 mice (Figure 2 and Figure S4).

In P4 blood, for the spleen and bone marrow of young adults, the percentage of CECs was similar for both genotypes, but *Tmcc2^−/−^* samples contained significantly fewer enucleated CECs and a high percentage of nRBCs. In contrast, the content of CECs in the knockout liver was increased, suggesting compensatory erythropoiesis (Figure 2E–L).

Fully mature *Tmcc2^−/−^* mice did not have skeletal defects characteristic for some human CDA types [7], nor abnormalities in the gross anatomy of the brain, spleen, liver, digestive tract, or heart. We observed no differences in gait or general demeanor that could suggest nervous system deficits. Normal numbers of CECs in the blood and the bone marrow and increased numbers in the spleen (Figure 2M–P) suggest that mature knockouts have active extramedullary erythropoiesis. The mature bone marrow and the spleen contained an increased fraction of polychromatic (stage III) CECs and decreased fractions of orthochromatic (stage IV) CECs and reticulocytes (stage V) (Figure 2Q–R), suggesting a partial maturation block or delay at the transition from polychromatic to orthochromatic erythroblast caused by a defect that is independent from the specific erythropoietic niche. Additionally, the total number of CECs in the adult spleen was significantly increased across all maturation stages, consistent with active extramedullary erythropoiesis, with the highest difference at the polychromatic stage (Figure 2S).

## 3. Discussion

Herein, we report that ablation of *Tmcc2* in mice causes an impairment of erythropoiesis in vivo. Leukocyte and megakaryocyte lineages (Figure S5) appeared unaffected. Persistent bone marrow phenotype in adult mice indicates that *TMCC2* plays a constitutive (rather than a purely developmental) role; thus, the underlying cellular cause of dyserythropoiesis does not subside with age, but rather extramedullary compensation or stringent control of RBC release from the bone marrow weaken the phenotype. Crucially, fewer mature erythrocytes and erythrocyte precursors (CECs) are enucleated in the knockouts in all erythropoietic tissues and ages tested, which raises the possibility that *TMCC2* participates in the acquisition of cellular competency for enucleation or in the process of enucleation itself. This notion is supported by a previously reported sharp increase in *TMCC2* expression in erythropoietic precursors at the polychromatic and orthochromatic stage, and by the observation that the *TMCC2* protein is differentially sorted between the maturing RBC and the pyrenocyte. In fact, over 95% of the *TMCC2* protein is expelled with the pyrenocyte [3], [Suppl mmc5.xlsx] during enucleation, and over 97% of *TMCC2* still present in the reticulocytes is lost during reticulocyte maturation [15], [Suppl2.xlsx]. The molecular details of the final steps of erythroblast maturation, including the removal of the nucleus and the clearance of the majority of organelles, are a subject of acute current interest [16]. Further mechanistic studies are required to explore the exciting possibility that *TMCC2* might play a role in these events. Additionally, a possible role of *TMCC2* in immunomodulation by CECs should be investigated [17,18].

A comparison of the phenotype of *Tmcc2^−/−^* mice and the results obtained in human primary erythroid culture system [4] is interesting, even though the technical differences between the two systems are likely more significant than the actual interspecies differences in the role of *TMCC2* in mice and humans. Two critical differences are that cells in culture are deprived of their natural erythropoietic niche and that the loss of *TMCC2* in knockdown experiments is sudden and potentially incomplete, while in the knockout mice, it is permanent and complete. While the observations from cultured erythroid precursors are highly valuable, they do not necessarily reflect the exact nature of the erythropoietic defect caused by the loss of *TMCC2* in vivo. Our knockout mice have a normal fraction of CECs in the erythropoietic organs, but the relative content of CECs at different stages of maturation is perturbed, which is a combination unlikely to be caused by a proliferation defect reported in the in vitro study. Perhaps the overall proliferation defect in *TMCC2* knockdown cultures might indeed be caused by increased cell lysis in the late stages of differentiation, as suggested by the authors of that study. We have not investigated the propensity of erythropoietic precursors to undergo apoptosis in our model; however, apoptosis of CECs maturing in their erythropoietic niche in vivo probably happens at a lower rate than in vitro. The disruption of cellular morphology is the most significant common observation in both models. It would be interesting to analyze the ultrastructure of the cultured *TMCC2* knockdown erythropoietic cells under the TEM and compare to our findings.

The practical value of our work for hematology is twofold. First, we describe a new murine model that can be used to study the mechanisms of erythropoiesis, neonatal anemia, and polycythemia in the adults. The presence of several features typically attributed to CDA types II–IV (Table S1) is interesting because animal models of CDAs are difficult to obtain, often due to subtle interspecies differences in gene expression or function [19].

Additionally, our study provides a possible explanation for why polymorphisms that affect *TMCC2* undergo negative evolutionary selection in humans [4]. While pathogenic variants of human *TMCC2* are currently unknown, polymorphisms such as variant C of intronic rs61823972 are associated with altered erythrocyte properties [20]. In our opinion, more deleterious *TMCC2* mutations will eventually be identified in patients who suffer from erythrocyte disorders of unexplained etiology, including CDAs. The absence of *TMCC2* in hematological genetic panels used in recent studies delays this moment [21,22]. The developmental change in the phenotype of the knockout mice should alert clinicians to a possible bone marrow dysfunction caused by a *TMCC2* mutation, even when the basic blood work is relatively normal.

Standard whole exome or whole genome sequencing (WES/WGS) analysis requires filtering of variants based not only on the quality of sequencing data but also on their estimated biological or clinical relevance [23]. Increased understanding of gene function can lead to improved sensitivity of detection of disease-causing variants through better filtration strategies for exome sequencing data [24,25]. Hence, translating our findings to clinical practice could be accelerated by a filtered retrospective analysis of the WES/WGS data previously obtained by others, focused on the *TMCC2* genetic variants in patients with various bone marrow failure conditions, especially in a cohort of CDA patients. The current Exome Variant Server [26] release contains 24 potentially damaging polymorphisms in the human *TMCC2* gene (NM_014858.3/NP_055673). In the region that overlaps with the erythroblast specific isoform (aa. 250 onwards), there are 12 “probably damaging” (V275M, A324T, R359G, R371W, G388S, A393T, S503N, R524W, A639V, K645R, V656M, and V705M) and 2 “possibly damaging” missense variants (V329M and Y510C). Most of these and additional damaging frame shift and premature termination mutations (D277MfsX21, P454RfsX23, S503AfsX38, I526GfsX50, D632X, and Y700X) are also reported in the Genome Aggregation Database, with allele count and frequency [27,28]. No homozygous carriers are known, but V275M is the most frequent, followed by A639V and V705M. Future identification of symptomatic carriers and additional human mutations in *TMCC2* is going to pose new questions and challenges regarding the effect that different truncations, amino acid substitutions, and copy number variations have on the molecular function of *TMCC2* and erythropoiesis. Additional mouse models might be required to resolve these issues; however, many molecular details will be addressed through the use of in vitro studies. Induced pluripotent stem cells (iPSCs) derived from CDA IV patients have been successfully used to elucidate the cellular impact of the disease causing mutation in KLF1 [29]. By analogy, such an approach could be used to study human *TMCC2* mutations.

## 4. Materials and Methods

Mice: *Tmcc2* knockout mice were generated using clustered regulatory interspaced short palindromic repeats (CRISPR)/CRISPR-associated protein 9 (Cas9)-mediated genome editing. Guide RNA (gRNA target sequence: 5′TGGCAGCCTTAGTTCGCTG3′) and Cas9 protein injection into the pronucleus of mouse oocytes was done commercially at the Mouse Genome Engineering Facility in Warsaw, Poland (https://crisprmice.eu/, accessed on 3 May 2022). Targeting was confirmed by PCR using primers flanking the deleted sequence (CC2gtF1 5′ACGTATAAAGGTCATGCCG3′, CC2gtR1 5′CCATTCTGCTCGATTTCCTT3′) and by sequencing. CRISPR/Cas9-mediated deletion of 11 nucleotides (c.835_845del11b) introduced a premature stop codon (ENSMUST00000045473.15 p.279X) that affects all known splice variants, including ENSMUST00000142609.7, which corresponds to the human erythroblast specific isoform ENST00000329800.7. Genotyping by PCR with Taq polymerase (VWR, #733-1820) and primers flanking the 11 bp deletion (nCC2gtF3: 5′TGACAAGGGAGATCTTGTGG3′, nCC2gtR3: 5′TGCTCGATCTTGATCTGCTC3′) yields 177 bp wildtype and 166 bp knockout bands that are resolved by electrophoresis on a 30 cm long, 3% agarose gel in 1 × TBE buffer, conducted at 105 V for at least 200 min. The line was generated on C57BL/6/Tar × CBA/Tar genetic background (Department of Biology UW), subjected to embryo transfer using C57BL/6 (Nencki Institute of Experimental Biology, Warsaw) and crossed to C57BL/6 before establishing an interbreeding colony at the animal facility of the Warsaw Medical University (CLZD WUM). Animals were housed in controlled environmental conditions in a specific pathogen-free (SPF) animal facility, with water and food provided ad libitum.

Sample collection: Mice were euthanized in accordance with institutional guidelines. Whole blood samples were collected immediately by cardiac puncture (neonatal mice) or from orbital sinus (adult mice). For complete blood count (CBC), whole blood was diluted to 1:5 in PBS and analyzed on a Sysmex XN-2000 hematology analyzer. To obtain serum samples, blood was collected in standard microcentrifuge tubes, left for 30 min to allow for clot formation, and separated by centrifugation. Biochemical analysis of the serum was performed using serum diluted to 1:5 in PBS using Cobas^®^ 8000 Roche modular analyzer, according to the manufacturer’s protocol. Bone marrow was obtained by centrifugation of dissected femur and tibia.

Bilirubin assay: Total bilirubin in serum from 20 P3 pups born in 3 litters (5 knockouts and 15 heterozygous controls) was measured using a colorimetric assay kit based on the Jendrassik–Grof principle (BioVision # K553-100), according to manufacturer’s instructions. Briefly, 30 µL of serum was collected from each mouse, mixed with 50% DMSO to a final volume of 100 μL, and divided into two equal aliquots of 50 µL in a flat bottom 96-well plate, thus forming a sample and a matched background control pair. Samples were incubated with 125 µL of complete reagent mix (20 µL of reagent 1, 5 µL of reagent 2, 100 µL of catalyst per sample) and control wells with a mix in which reagent 2 was replaced with water. After 30 min incubation at room temperature, 75 µL of total bilirubin probe was added to each well, and the plate was incubated for an additional 20 min and analyzed on a microplate reader. A standard curve with 0, 0.25, 0.5, 1, 2, and 4 μg of bilirubin/well was prepared in duplicate on the same plate as the serum samples. Total and direct bilirubin concentration in samples from adult mice was determined with the Cobas^®^ 8000 Roche modular analyzer.

Electron microscopy: P2 blood samples were fixed in 2.5% Glutaraldehyde and 1.5% Paraformaldehyde in 0.1M cacodylate buffer at RT for 1 hour, centrifuged at 3200 RPM for 10 min, washed three times with cacodylate buffer, treated with 1% OsO_4_ solution for 1 h at 4 °C, and cells were dehydrated by ethanol solutions of increasing concentration (30%, 10 min; 50%, 10 min; 70%, 24 h; 90%, 10 min; 96%, 10 min; anhydrous EtOH, 10 min; and finally, acetone, 10 min). After fixation and dehydratation, the cells were embedded into epone resin (first mixed with successive acetone solutions of increasing epone concentration: 1:3, 30 min; 1:1, 2 h; 3:1, 5 h; and then pure for 12 h). After saturation, the epone was polymerized in blocks in 60 °C for 24 h in an incubator (Agar Scientific, Stansted, UK). The polymerized samples were cut into ultrathin sections (70 nm thick) with an RMC MTX ultramicrotome (Boeckeler Instruments, Tucson, AZ, USA), placed onto copper grids, contrasted with uranyl acetate and Reynolds reagent (Reynolds, 1983), and analyzed with a LIBRA 120 transmission electron microscope (Carl Zeiss, Oberkochen, Germany) at 120 keV. Photographs were made with a Slow-Scan CCD camera (ProScan, Graben, Germany), using the EsiVision Pro 3.2 software (Soft Imaging Systems GmbH, Münster, Germany).

Flow Cytometry: Flow cytometry was performed on Fortessa LSR (BD Biosciences, Franklin Lakes, NJ, USA) operated by FACSDiva software. For data analysis, Flow Jo v10.6.1 software FlowJo, Ashland, OR, USA) was used, followed by statistical analyses with Graphpad Prism 8.4.3 (GraphPad Software, San Diego, CA, USA). Whole blood, liver, spleen, and bone marrow tissues were isolated from mice and mechanically dispersed by pressing gently through a filter cap of a collection tube using a rubberized 1 mL syringe piston. Liver tissues were enzymatically dispersed with 100 U/mL collagenase and 125 U/mL DNAse mix for 45 min at 37 °C before mechanical dispersion. For cell surface staining, cells were stained with Zombie NIR™ Fixable Viability Kit (BioLegend, San Diego, CA, USA), blocked on ice with 5% normal rat serum in FACS buffer (PBS; 1% BSA, 0.01% sodium azide), and then incubated for 30 min on ice with fluorochrome-labelled antibodies. Fluorophore-conjugated antibodies specific for mouse cell-surface antigens were as follows: anti-CD71 (clone R17217, cat. 46-0711-82, eBioscience, San Diego, CA, USA), anti-TER119 (clone TER-119, cat. 116208 BioLegend), and anti-CD44 (clone IM7, cat. 103019, BioLegend). To stain the nuclei, cells were incubated with Hoechst 33342 at a dilution of 1:10,000 in PBS. After washing in FACS buffer, cells were immediately analyzed.

Blood smear staining: A drop of blood was distributed over the slide and briefly air-dried. Cells were fixed with 70% ethanol for 5 min, stained with Mayer’s hematoxylin (VWR #10047005) for 10 min rinsed for 10 min under tap water, counterstained with Eosin Y (VWR #10047001) for 5 min, and finally washed and dehydrated in a series of 50%, 75%, 90%, and 100% ethanol. Stained blood smears were viewed without mounting under the Nikon Eclipse upright microscope with Nikon Plan Fluor 20×/0.5 or 40×/0.75 air objectives. Images were captured at a resolution of 1600 × 1200 pixels, 24 bit color, with the QImaging Retiga 2000R Fast 1394 camera in RGB mode controlled by Image-Pro Plus (Media Cybernetics Inc. version 7.0.1.658 WinXP/Vista). Images used for automated quantification were all taken with the 20× objective and 250 ms exposure time and analyzed in ImageJ/Fiji.

Cell transfection: HEK293 cells were grown on a 10 cm cell culture plate in DMEM supplemented with Penicillin/Streptomycin/Glutamine until ~70% confluent, and transfected with 5 µg of plasmid DNA containing a full length mouse *Tmcc2* cDNA clone (#MR210162, OriGene, in which the DDYK tag had been replaced by a C-terminal HA tag) in the presence of 15 uL of XtremeGene HP reagent (#06366236001, Roche). Cells were grown for an additional 16 h and lysed.

Western blotting: Protein extracts were prepared in 0.5% Triton X-100 in 50 mM Tris-Cl pH 7.5 with protease inhibitor cocktail (Complete tabs, EDTA-free # 11697498001, Roche). Extracts were mixed with 2× sample buffer with 5% β-mercaptoethanol and incubated at room temperature for 15 min. Samples were subjected to SDS-PAGE on a 10% TGX gel (#161-0183, Bio-Rad, Hercules, CA, USA), with Precision Plus Protein Dual Color Standard (161-0394SP, Bio-Rad). Proteins were transferred onto a 0.45 µm PVDF membrane (Hybond #10600023, Amersham, UK). Membranes were blocked with 5% non-fat milk or 5% BSA in TBS with 0.1% Tween and incubated overnight with the anti-*TMCC2* Rabbit Polyclonal antibody (25042-1-AP, Proteintech, Rosemont, IL, USA) [30] diluted to 1:2000 in 2.5% milk, followed by three washes with TTBS and 1 h incubation with HRP coupled anti-Rabbit secondary antibody (A0545-1ML, Sigma, St. Louis, MO, USA) diluted to 1:50000. Membranes were washed three times in TBS and incubated with ECL Plus reagent (GERPN2236, Sigma). Chemiluminescence images with exposures ranging from 5 s to 5 min were taken with Amersham Imager 600.

Statistical analysis: Statistical analysis and data plotting were performed in Microsoft Excel with the Real Statistics add-on or in GraphPad Prism 8.4.3 (GraphPad Software). Statistical significance of observed effects was assessed using parametric (unpaired Student t-test) or nonparametric (Mann–Whitney) tests, as indicated in figure legends.

## Data Availability

The data that support the findings of this study are available from the corresponding author upon reasonable request.

## Supplementary Materials

The following supporting information can be downloaded at https://www.mdpi.com/article/10.3390/ijms23095263/s1.
