# Supplementary material for: Ablation of Tmcc2 Gene Impairs Erythropoiesis in Mice
Source: Int J Mol Sci. 2022 May 9;23(9):5263. doi: 10.3390/ijms23095263 (PMC9102901; doi:10.3390/ijms23095263)
Supplement: Supplementary file 1 [file ijms-23-05263-s001.zip › ijms-1685892-supplementary.pdf]

Supplementary data

Kumari et al, 2022 Ablation of *Tmcc2* gene impairs erythropoiesis in mice

Figure S1

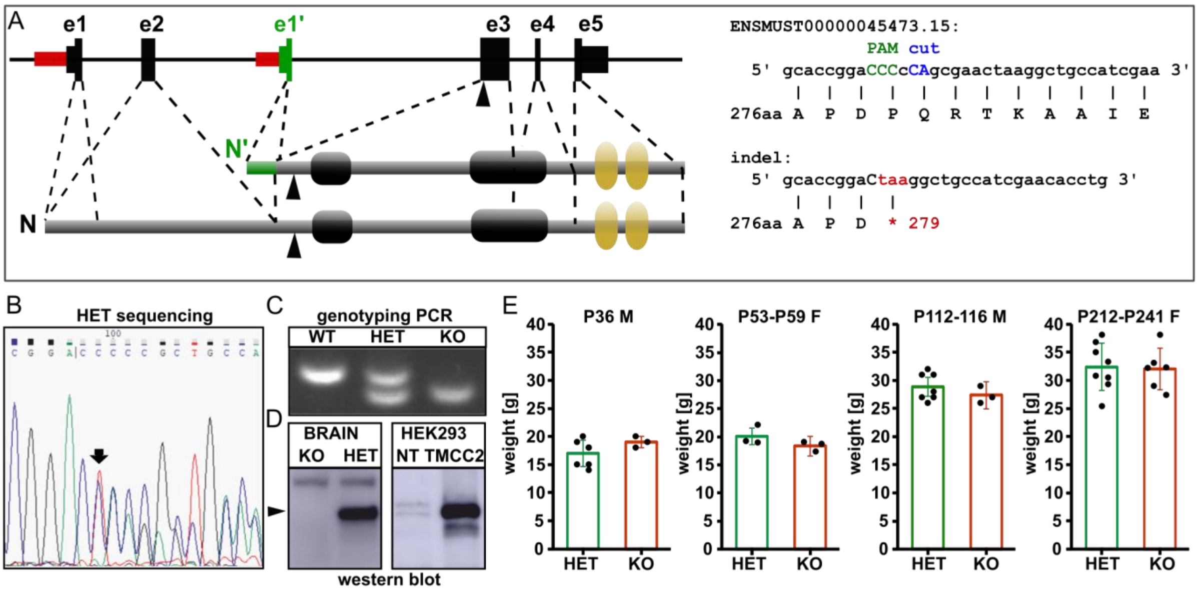

Figure S1. *Tmcc2* knockout mouse design. (A) Genomic structure of the *Tmcc2* gene (top) and domain structure of the main protein products resulting from transcription starting at alternative promoters (bottom). Exons are marked in black, the alternative first coding exon is green, and the promoters are depicted as red rectangles. The short isoform corresponding to the human erythroblast specific form starts at exon 1' (e1'). All isoforms share two coiled-coil regions (black cylinders) and two transmembrane domains (gold cylinders) but differ in respect to the N-terminal segment that harbors the binding site of the 25042-1-AP antibody.[1] CRISPR/Cas9 mediated deletion of 11 nucleotides introduces a premature stop codon that truncates the polypeptide chain before the first coiled-coil domains in all isoforms. (B) Validation of the deletion by Sanger sequencing of a PCR products obtained from the heterozygous founder genomic DNA with primers flanking the target sequence. (C) Genotyping PCR of mouse tail DNA samples yields wild-type (177 bp) and knockout (166 bp) bands. (D) Specific ~80 kDa TMCC2 band is absent in *Tmcc2*$^{-/-}$ whole brain extract western blots, unspecific ~130 kDa band is present in both genotypes. The antibody recognizes the recombinant TMCC2 protein band of expected size in transfected HEK293 cells. (E) Body weight of adult *Tmcc2*$^{-/-}$ mice is comparable to that of sex and age matched heterozygous controls from the same colony: 1-month-old males (N_{HET}=6, N_{KO}=3), 2-month-old females (N_{HET}=3, N_{KO}=3), 3-4-month-old males (N_{HET}=6, N_{KO}=3), and 7-8-month-old females (N_{HET}=8, N_{KO}=6).

Figure S2

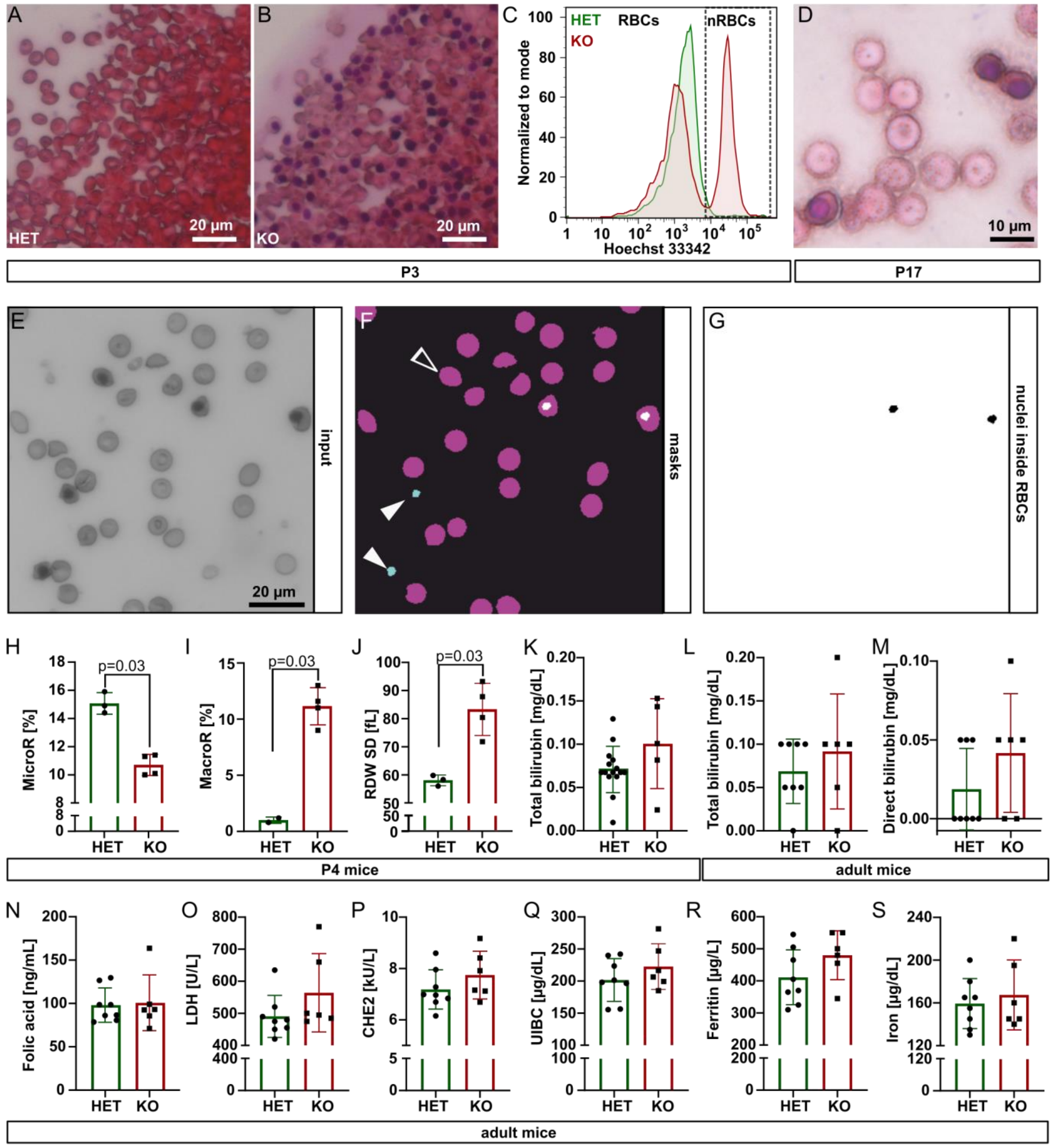

Figure S2. Properties of *Tmcc2*$^{-/-}$ red blood cells and biochemical parameters related to anemia. (A-B) Whole blood smears of P3 mice. (C) Representative Hoechst 33342 fluorescence intensity of RBCs in the spleen of P4 mice. (D) nRBCs in the blood of P17 mice prompt further investigation of adult erythropoiesis. (E-G) Principle of quantitative analysis of nucleated red blood cells (nRBC) in blood smears (related to Figure 1J). (E) Example of an input image. (F) Masks corresponding to individual cells and nuclei were generated independently and overlaid. Nuclei that belong to clumped cells (full arrowheads) were rejected to avoid overestimation, while occasional nuclei escaped detection (empty arrowhead), leading to a modest underestimation of the actual nRBC fraction. (G) Only nuclei that belong to cells scored as single were taken into account. Analysis was performed on a set of 151 microscope images, between 12 and 18 per each of the 5 knockout and 5 heterozygous control mice. A total of 30698 single cells, 13680 in the *Tmcc2*$^{-/-}$ and 17018 in control samples, and 717 nuclei that were inside these cells were counted. A percentage of nRBCs was calculated independently for each animal. Scale bar in (E) to 20 µm and applies to (E-G). (H-J) Anemia in P4

mice (N_{HET}=3, N_{KO}=4) is macrocytic. (H) Percentage of microcytes (MicroR), (I) percentage of macrocytes (MacroR), (J) Red blood cell distribution width standard deviation (RDW SD). (K) Bilirubin concentration in serum is normal in P4 (either sex) and (L-M) mature (7-8-month-old, female) mice (N_{HET}=8, N_{KO}=6). (E-L) Main biochemical parameters used in the diagnosis of anemia: (N) folic acid, (O) lactate dehydrogenase (LDH) activity, (P) pseudocholinesterase (CHE2) activity, (Q) unsaturated iron-binding capacity (UIBC), (R) ferritin, and (S) iron. Serum biochemistry of adult (female) samples performed with Cobas® 8000 Roche modular analyzer. Data presented as mean ± SD. (H-J) *P*-values by the Mann-Whitney test and are the same in (H-J) since all the knockout values differ from all the control values in the same direction and only the ranks, not the actual values, influence the result of this test. (K-S) Statistical significance was assessed with a t-test, no significant differences found.

Figure S3

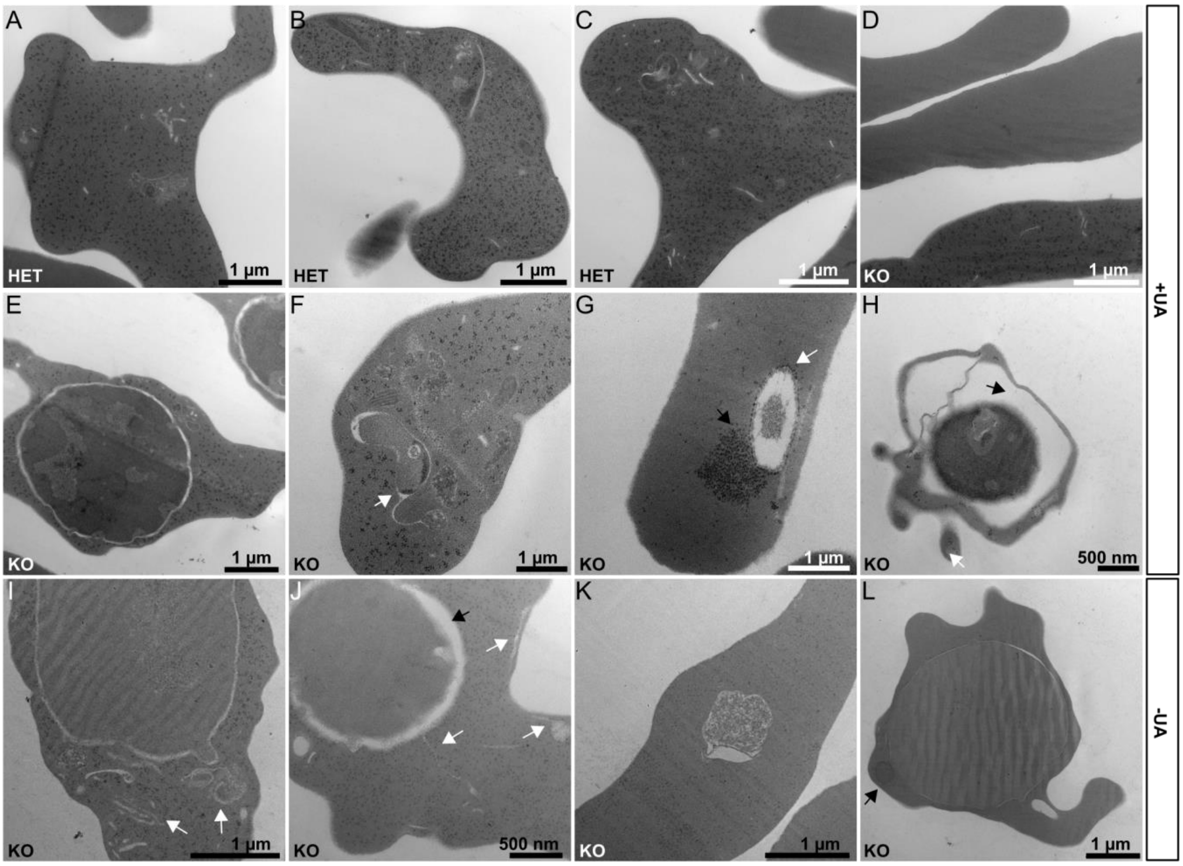

Figure S3. Additional TEM images. Representative control (A-C) and *Tmcc2*$^{-/-}$ (D-L) reticulocytes and nRBCs from P2 blood (related to Figure 1K-T). (D) Two mature *Tmcc2*$^{-/-}$ RBCs next to a reticulocyte. (E) Two juxtaposed *Tmcc2*$^{-/-}$ nRBCs with numerous ribosomes. (F) Reticulocyte with an autophagosome and remnants of organelles. (G) Ribosomes grouped next to a vacuole-like structure (white arrow) that is decorated by regularly interspaced ribosomes. (H) A putative pyrenocyte. (I-J) Multiple membranous cisterns and canaliculi (white arrows), dilated perinuclear space black arrow). (K) Nearly mature reticulocyte with a central autophagosome. (L) Nucleated RBC with clear cytoplasm and a high nucleus/cytoplasm ratio. Images (A-H) come from grids contrasted with Reynolds reagent and uranyl acetate, uranyl acetate was skipped (-UA) in (I-L). Scale bars represent 1 µm (A-G, I, K-L), and 500 nm (H, J).

Figure S4

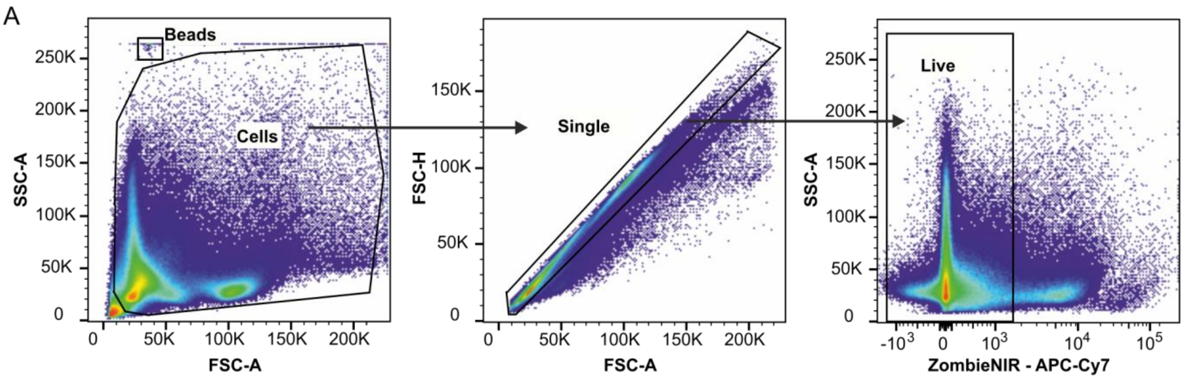

Figure S4. Gating strategy for flow cytometry. (A) Gating strategy used to define single live cells, directly preceding gating steps in the main Figure 2C-D. ZombieNIR-APC-Cy7 dye stains dead cells that are excluded from subsequent analyses.

Figure S5

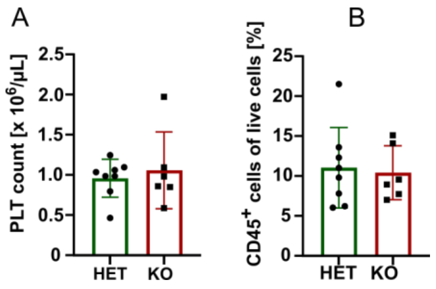

Figure S5. Non-erythropoietic lineages do not appear to be affected in mature *Tmcc2*$^{-/-}$ mice. (A) Platelet (megakaryocyte lineage) count (PLT) in the whole blood. (B) Relative abundance of $CD45^{+}$ cells (lymphocyte lineage) among live cells in the spleen. Data presented as mean ± SD, (N_{HET}=8, N_{KO}=6, female). Statistical significance was assessed with a t-test.

Supplementary Literature

1. Cifre M, Palou A, Oliver P. Cognitive impairment in metabolically-obese, normal-weight rats: identification of early biomarkers in peripheral blood mononuclear cells. Mol Neurodegeneration. 2018;13:14.

Table S1. Comparison of murine *Tmcc2*$^{-/-}$ phenotypic features to known human CDA types.

Feature	CDA I	CDA II	CDA III	CDA IV and XLTDA	*Tmcc2* knockout
Mutated gene(s)	*CDAN1* and *CDIN1*	*SEC23B*	*KIF23*	*KLF1*, *GATA1*, unknown	*Tmcc2*
Anemia	Macrocytic	Normocytic	Normocytic or macrocytic	Normocytic	Macrocytic, compensated normocytic
Morphology of erythroblast	Internuclear chromatin bridges between the nuclei pairs of intermediate erythroblasts	Binuclearity, multinuclear mature erythroblasts	Giant multinucleated erythroblasts in the bone marrow	CDA I–like, CDA II–like, others	Numerous nRBCs in young and adult, multinucleated (2-4 nuclei) and rare giant multinucleated (4-12 nuclei) erythroblasts circulating in newborns
Electron microscopy features	Dense heterochromatin, demarcated clumps with small translucent vacuoles, “spongy” or “Swiss cheese” appearance of the nucleus	Peripheral cisternae beneath the plasma membrane, a discontinuous double membrane	Clefts in heterochromatin, autophagic vacuoles, intranuclear cisternae	Atypical cytoplasmic inclusions, invagination of the nuclear membrane, and marked heterochromatin	Invagination of the nuclear membrane, cytoplasmic intrusions in the nucleus, peripheral cisternae beneath the plasma membrane, discontinuous double membrane, dilated perinuclear space

Table S2. Summary of CBC parameters presented in graphs.

CBC parameter	Tmcc2$^{+/-}$ (HET) Mean±SD	Tmcc2$^{-/-}$ (KO) Mean±SD	Figure
Pups (P3-P4)			
RBC [x 10^6/µl]	2.90±0.17	2.05±0.09	Figure 1U
HCT [%]	23.33±1.89	19.25±0.96	Figure 1U
HGB [g/dl]	8.00±0.87	5.00±0.41	Figure 1U
MCH [fmol/cell]	27.53±1.27	24.40±2.02	Figure 1U
MCV [fL/cell]	80.50±1.75	93.90±1.40	Figure 1U
nRBC [x 10^6/µl]	0.17±0.03	291.01±29.55	Figure 1U
MicroR [%]	15.07±0.76	10.70±0.75	Figure S2H
MacroR [%]	0.90±0.26	11.15±1.66	Figure S2I
RDW SD [fL]	58.03±1.91	83.3±9.29	Figure S2J
Adult mice			
RBC [x 10^6/µl]	11.08±0.90	16.65±1.91	Figure 1V
HCT [%]	51.26±4.77	78.07±8.82	Figure 1V
HGB [g/dl]	15.48±1.34	23.07±2.54	Figure 1V
MCH [fmol/cell]	14.11±0.36	13.88±0.26	Figure 1V
MCV [fL/cell]	46.23±1.28	46.93±0.79	Figure 1V
nRBC [x 10^6/µl]	0.01±0.03	6.67±2.90	Figure 1V
PLT [x 10^6/µl]	0.96±0.24	1.06±0.47	Figure S5A
